# Mortality following the Haitian earthquake of 2010: a stratified cluster survey

**DOI:** 10.1186/1478-7954-11-5

**Published:** 2013-04-25

**Authors:** Shannon Doocy, Megan Cherewick, Thomas Kirsch

**Affiliations:** 1 Johns Hopkins Bloomberg School of Public Health, 615 N Wolfe St, Ste E8132, Baltimore, MD, 21205, USA; 2 Johns Hopkins School of Medicine, Baltimore, MD, USA

**Keywords:** Haiti, Earthquake, Mortality, Natural disaster

## Abstract

**Introduction:**

Research that seeks to better understand vulnerability to earthquakes and risk factors associated with mortality in low resource settings is critical to earthquake preparedness and response efforts. This study aims to characterize mortality and associated risk factors in the 2010 Haitian earthquake.

**Methods:**

In January 2011, a survey of the earthquake affected Haitian population was conducted in metropolitan Port-au-Prince. A stratified 60x20 cluster design (n = 1200 households) was used with 30 clusters sampled in both camp and neighborhood locations. Households were surveyed regarding earthquake impact, current living conditions, and unmet needs.

**Results:**

Mortality was estimated at 24 deaths (confidence interval [CI]: 20–28) per 1,000 in the sample population. Using two approaches, extrapolation of the survey mortality rate to the exposed population yielded mortality estimates ranging from a low of 49,033 to a high of 86,555. No significant difference in mortality was observed by sex (p = .786); however, age was significant with adults age 50+ years facing increased mortality risk. Odds of death were not significantly higher in camps, with 27 deaths per 1,000 (CI: 22–34), compared to neighborhoods, where the death rate was 19 per 1,000 (CI: 15–25; p = 0.080). Crowding and residence in a multistory building were also associated with increased risk of death.

**Conclusions:**

Haiti earthquake mortality estimates are widely varied, though epidemiologic surveys conducted to date suggest lower levels of mortality than officially reported figures. Strategies to mitigate future mortality burden in future earthquakes should consider improvements to the built environment that are feasible in urban resource-poor settings.

## Introduction

Urbanization increases disaster risk because of the high concentrations of population and assets that are vulnerable when a major disaster occurs. In lower- and middle-income countries, continual social and demographic change also complicate issues of urban disaster risk management. Concentrations of economically poor slum residents, especially in cases where slum settlements expand to hill slopes or flood plains, increase the hazard profile of the city. Lack of urban planning capacity, implementation, and enforcement of building codes and poorly regulated development are common problems across many urban centers in lower- and middle-income countries that increase disaster risk [1] In Haiti, the capital of Port-au-Prince is known to be at risk for hydro-meteorological hazards, in particular hurricanes, with little concern for geologic hazards because of the lack of significant historical earthquakes [2]. This changed with the magnitude 7.0 earthquake that struck on January 12, 2010, and devastated the city with a reported 222,750 deaths, 300,000 injured, 1.5 million displaced, and more than 3 million affected. Relief and recovery operations were historic, and by October 2010, the response was one of the largest in history with an estimated cost of $4.5 billion [3]. This study sought to estimate mortality from the 2010 Haiti earthquake and identify risk factors for mortality in major earthquakes in low-income settings.

## Methods

A cross-sectional cluster survey of the earthquake-affected population in metropolitan Port-au-Prince was conducted in January 2011 to assess the impact of the 2010 earthquake on Haitian households and to characterize perceptions of humanitarian assistance at one year postearthquake; the mortality data presented in this paper were collected as part of this larger study. The proportion of the affected populations residing in camps and neighborhoods could not be accurately estimated from available government and United Nations information; thus, a stratified design was used to enable comparison between camp and noncamp (neighborhood) populations. A stratified cluster survey with 60 clusters of 20 households, including 30 camp and 30 neighborhood clusters, was used to characterize earthquake impact and receipt of humanitarian assistance in camp and neighborhood populations. Sample size was calculated based on the broader study objective of comparing the status of camp and noncamp populations. Calculations were based on the most conservative prevalence rate of 50% and a hypothesized difference of 10% between camp and noncamp populations for measures of impact, inadequate living conditions, and receipt of humanitarian assistance; other parameters included 80% power, alpha = 0.05, and an anticipated cluster sample design effect of 1.5. Households were included in the survey only if the dwelling had been damaged, income or livelihood affected, or a household member was injured or died as a result of the earthquake. The survey instrument was developed in English and translated into Creole and conducted by trained Haitian nationals.

Clusters were assigned using probability proportional to size (PPS) sampling. Camps were sampled using the PPS methodology from a list of planned and spontaneous settlements obtained from the Camp Coordination and Management Cluster [4]. The starting point in each camp cluster was a randomly selected intersection, and every third shelter was sampled until 20 households were completed. For neighborhoods, cluster allocation was based on a remote sensing building damage assessment [5], under the assumption that the number of moderately and heavily damaged residential structures was proportional to the earthquake-affected population within an administrative unit. The proportion of moderately to heavily damaged residential buildings from the damage assessment and 2009 population estimates were used to estimate the affected population within each commune (similar to a district) in Port-au-Prince. Clusters were then assigned to communes proportionate to the estimated affected population. Within each commune, clusters were assigned to sections (similar to a subdistrict or neighborhood) proportionally based on 2009 population estimates [6]. In each section, geographic coordinates were randomly selected and the nearest intersection was used as the cluster start. Then, a randomly selected number and direction were generated among the streets or pathways meeting the intersection to select one for the survey start location. From there, every third residential entrance was sampled; in buildings with multiple households, one household was randomly sampled. Data were collected using questionnaire-based interviews by a team of local Haitian interviewers from Port-au-Prince. Any adult household member was eligible to serve as a respondent; however, the head of household usually acted as the respondent if he or she was present at the time of the interview. If absent, second priority was given to the head of household’s spouse or the caretaker of young children. Only pre-earthquake household members were used for mortality estimation; individuals that joined the household after the earthquake were excluded because they were originally part of other households, and a separate interview would have been needed. This would have been difficult given that many of them were children. The response rate was high (>95%) in households in which adults were present; the number of households in which no adult was present and those that did not meet inclusion criteria were not recorded.

Data analysis was performed with Stata version 12 (College Station, TX) using simple logistic regression, multivariable logistic regression, and mixed-effects logistic modeling methods, as well as chi-squared and t-tests. The design effect was calculated at 1.21; consequently, odds of mortality and related confidence intervals (CIs) were adjusted to reflect the cluster survey design. Multiple variable logistic regressions were conducted with a mixed-effect model (*xtmelogit* command of Stata). Mixed-effects simple and multiple logistic regressions were used to measure the total unadjusted and adjusted odds of mortality. The final model was selected based on Akaike Information Criterion and Pearson’s goodness of fit test.

The study was certified exempt by the Johns Hopkins Bloomberg School of Public Health Institutional Review Board and approved by the Haitian Ministry of Public Health and Population. This work was supported by the Johnson and Johnson Foundation and the Johns Hopkins Bloomberg School of Public Health Center for Refugee and Disaster Response.

## Results

In January 2011, when the survey was conducted, the 1,197 households surveyed had a combined population of 6,696 individuals, of which 6,547 (97.9%) were reported as household members on the day of the earthquake. A total of 149 individuals were born or moved into households after January 12, 2010 and were not included in the earthquake-exposed population that served as a denominator for mortality calculations. Descriptive characteristics of the sample population are summarized in Table 1. The average household size was 5.3, and household size was similar in the camp and neighborhood populations (p = 0.474). The population was 52.5% female, and camps had a significantly higher proportion of females than neighborhoods (53.9% female vs. 51.0% female; p = 0.017). Age composition of the sample population was as follows: 0–17 years, 35.2%; 18–49 years, 56.44%; 60+ years 8.39%. The average age was 26.1 years (standard deviation [SD] = 16.1), and camp residents were significantly younger than neighborhood residents (23.8 years vs. 27.4 years; p < 0.001). The highest educational level attained by any household member (categorized as none, primary school or less, secondary school, or higher education) showed significant differences between camps and neighborhoods, with camp residents having lower education levels (p < 0.001). As compared to neighborhood residents, camp residents were significantly less likely to own their own home (53.2% vs. 22.7%) and more likely to live in crowded conditions (24.9% vs. 39.1%, defined as ≥4 people/room) prior to the earthquake, suggesting that households of lower socioeconomic status were more likely to be displaced.

**Table 1:** Comparison of baseline characteristics by postearthquake residence location

	***Neighborhoods***		***Camps***		***Total***		***p-value***
	***(n = 3261)***		***(N = 3286)***		***(N = 6547)***		
**Baseline characteristics of the sample population**							
**Age**	**n**	**(%)**	**n**	**(%)**	**n**	**(%)**	**<0.001**^**¥**^
0-17	1010	30.1	1293	39.5	2303	35.2	
18-49	1920	58.88	1775	54.02	3990	60.9	
50+	331	10.15	218	6.63	230	3.5	
Mean (SD)	27.4(16.9)	23.8 (17.0)	25.4(16.7)				
**Sex**							**0.017**^**¥**^
Males	1567	48.1	1490	45.3	3057	46.7	
Females	1632	50.1	1748	53.2	3380	51.6	
**Household education**							**<0.001**^**Ω**^
None	76	2.3	217	6.7	293	4.5	
Primary school or less (%)	428	13.2	811	25.0	1239	19.1	
Secondary school (%)	1764	54.4	1925	59.2	3689	56.8	
Higher education	976	30.1	298	9.2	1274	19.6	
**Pre-earthquake living conditions**							
**Type of house**							**0.219 **^**Ω**^
Detached single family	1232	37.9	1233	37.5	2465	37.7	
Attached single family	1497	46.0	1463	44.5	2960	45.3	
Apartment/multiple dwelling	525	16.1	590	18.0	1115	17.1	
**Multilevel building**							**0.249**^**¥**^
Single level	2099	67.0	1901	65.5	4000	66.2	
Multiple levels	1039	33.0	1002	34.52	2041	33.8	
**Wall material**							**0.291**^**¥**^
Concrete or brick	3072	94.9	3136	95.4	6208	95.2	
Other	166	5.1	150	4.6	316	4.8	
**Roof material**							**0.342 **^**Ω**^
Cement/concrete	2014	61.8	2014	61.3	4028	61.5	
Metal sheeting	1193	36.6	1272	38.7	2465	37.7	
Plastic/thatch	54	1.7	0	0.0	54	0.8	
**Home and land occupancy**							**<0.001**^**Ω**^
Own home w/ title to land	1731	53.2	741	22.7	2472	37.9	
Own home w/o title to land	274	8.4	182	5.6	456	7.0	
Rent	1244	38.2	2322	71.0	3566	54.6	
Other	8	0.3	25	0.8	33	0.5	
**Crowding category**							**<0.001 **^**Ω**^
<2.0	1248	38.3	679	20.7	1927	29.4	
2.0-2.9	825	25.3	744	22.6	1569	24.0	
3.0-3.9	376	11.5	577	17.6	953	14.6	
4.0+	812	24.9	1286	39.1	2098	32.1	

¥ Calculated using the t-test.

Ω Calculated using analysis of variance.

**Figure 1:**
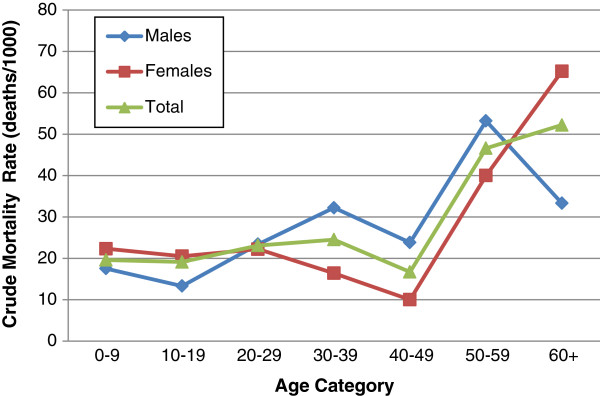
Age and sex specific morality rates.

A total of 159 deaths were reported in the pre-earthquake population between the January 12, 2010 earthquake and the January 2011 survey (of 6,536 individuals with reported survival status). Of these, 153 were direct deaths that occurred on the day of the event (95%) or within a month as result of earthquake-sustained injuries (5%). The overall earthquake mortality rate is estimated to be 24 deaths/1,000 (CI: 20–28) (Table 2). Elevated mortality was observed among households residing in camps, at 28 deaths/1,000 (CI: 22–34), as compared those in neighborhoods where the death rate was 19 deaths/1,000 (CI: 15–25) (p = 0.030). Age- and sex-specific mortality rates are presented in Figure 1. No significant difference in mortality was observed between males and females (p = 0.786). When assessed by age, mortality rates were as follows: children 0–17 years, 16 deaths/1,000 (CI: 11–22); adults 18–49 years, 24 deaths/1,000 (CI: 20–31); older adults 50+ years, 44 deaths/1,000 (CI: 28–64).

**Table 2:** Mortality estimates and odds of death by individual and household characteristics

	**Total exposed**	**Total deaths**	**Mortality rate (deaths/1000)**		**Odds of death**^**§**^		
			**Rate**	**95% CI**	**Odds ratio**	**95% CI**	**p-value**
***Overall***	6383	153	23.97	20.36- 28.03			
***Sex***							
Male	3052	71	23.26	18.21 – 29.25	Reference		
Female	3379	76	22.49	17.76 – 28.07	0.96	0.71 – 1.33	0.786
***Age category***							<0.003*
0-17	2299	37	16.09	11.36 – 22.11	Reference		
18-49	3689	92	24.93	20.15 – 30.49	1.56	1.06 – 2.30	0.023
50+	548	24	43.79	28.26 – 64.47	2.80	1.66 – 4.72	<0.001
***Education level***							0.399*
None	289	11	38.06	19.15 – 67.08	Reference		
Primary	1237	27	21.83	14.43 – 31.60	0.59	0.28 – 1.24	0.165
Secondary	3685	83	22.52	17.98 – 27.85	0.61	0.31 – 1.20	0.150
Higher education	1273	31	24.35	16.69 – 34.39	0.62	0.29 – 1.32	0.213
***Crowding***							0.082*
<2.0	1927	35	18.16	12.68 – 25.17	Reference		
2.0-2.9	1566	37	23.63	16.69 – 32.42	1.32	0.82 – 2.13	0.257
3.0-3.9	949	23	24.24	15.42 – 36.15	1.28	0.74 – 2.23	0.375
4.0+	2094	58	27.70	21.10 – 35.66	1.52	0.97 – 2.37	0.069
***Current cocation***							
Neighborhood	3258	63	19.34	14.89 – 24.67	Reference		
Camp	3278	90	27.46	22.13 – 33.64	1.44	0.90 – 2.29	0.127
***Multilevel***							
1 level	3999	76	19.00	15.00 – 23.73	Reference		
>1 level	2031	67	32.99	25.66 – 41.71	1.98	1.38 – 2.83	<0.001

§ Mixed-effect logistic modeling was used to obtain odds ratio and CI estimates accounting for cluster design.

*Indicates overall p-value for the categorical variable calculated using the likelihood ratio test.

Variables related to socioeconomic status, such as highest household education level attained, whether or not the pre-earthquake home had multiple levels, and crowding, were associated with mortality outcomes. The highest mortality rate was found among households in which no member had completed primary school (38 deaths/1,000, CI: 19–67), although as a categorical variable, household education was not significant in predicting mortality. Crowding can serve as a proxy for socioeconomic status and was associated with increased mortality risk; however, this finding was of marginal statistical significance (p = 0.082). Residence in a multistory building was associated with increased risk of death; mortality rates among households in single and multistory residences were 19 deaths/1,000 (CI: 15–24) and 33 deaths/1,000 (CI: 26–42) (p < 0.001).

In simple logistic regression, age and housing characteristics were significantly associated with increased mortality risk (Table 3). In the multiple logistic regression model, age and housing characteristics remained significantly associated with increased odds of death. As compared to children 0–17 years, middle-age (18–49 years) and older (50+ years) adults were 1.34 (CI: 0.89-2.01) and 2.50 (CI: 1.41-4.39) times more likely to experience death, respectively. Members of households in residing in multistory buildings had a mortality risk of 1.98 (CI: 1.38-2.83) times greater than residents of single level buildings. Crowding was a marginally significant predictor of mortality (p = 0.082) but was included in the final model based on log rank tests. Within category comparisons indicated that odds of death were 1.7 times (CI: 1.06-2.69) higher in populations with more than four people per room as compared to populations with two or fewer people per room (p = 0.027). The mortality rate among populations displaced to camps was 1.5 (CI: 0.95-2.38) times greater than populations in neighborhoods; however, this finding was only marginally significant (p = 0.080).

**Table 3:** Crude and adjusted Odds ratios for mortality

	**Crude odds**			Adjusted odds**		
	**Odds ratio**	**95% CI**	**p-value**	**Odds ratio**	**95% CI**	**p-value**
**Age category**						<0.003*
0-17 (reference)	1.00	1.06 – 2.30	0.023	1.34	0.89 – 2.01	0.163
18-49	1.56		<0.001	2.50	1.41 – 4.39	0.002
50+	2.80	1.66 – 4.72				
**Multilevel**						
Single level (reference)	1.00					
Multiple levels	1.76	1.26	0.001	1.98	1.38 – 2.83	<0.001
**Crowding category**						0.082*
<2.0 (reference)	1.00					
2.0-2.9	1.32	0.81 – 2.13	0.257	1.34	0.81 – 2.20	0.252
3.0-3.9	1.28	0.74 – 2.23	0.375	1.31	0.73 – 2.34	0.373
4.0+	1.52	0.97 – 2.37	0.069	1.69	1.06 – 2.69	0.027
**Current residence location**						
Neighborhood (reference)	1.00					
Camp	1.44	0.90 – 2.29	0.127	1.50	0.95–2.38	0.080
***Pre-earthquake living conditions***						
**Home Owner**	1.00		0.222*			
Own home w/ title (reference)	1.49	0.85 – 2.61		-	-	-
Own home w/o title	0.92	0.65 – 1.29	0.167	-	-	-
Rent	-		0.616-	-	-	-
Other				-	-	-
**Housing type**			0.003*			
Detached single family home	1.00	-	-	**-**	**-**	**-**
Attached single family home	0.93	0.64 – 1.37	0.720	**-**	**-**	**-**
Apartment or multifamily dwelling	1.99	1.33 – 2.99	0.001	**-**	**-**	**-**
**Wall material**						
Cement/concrete	1.00	-	-	**-**	**-**	**-**
Other	1.24	0.63 – 2.46	0.536	**-**	**-**	**-**
**Roof material**			0.097*			
Concrete/brick	1.00	-	-	**-**	**-**	**-**
Metal sheeting	0.68	0.48 – 0.97	0.032	**-**	**-**	**-**
Plastic/thatch	0.69	0.10 – 5.04	0.714	**-**	**-**	**-**

*Indicates overall p-value for the categorical variable using the likelihood ratio test.

** E [log odds of death] = ß_0_ + ß_1_i.age_cat3 + ß_2_multilevel + ß_3_crowd_cat + ß_4_location ; Pearson’s goodness of fit test: χ^2^ = 94.22, p = 0.280.

Mortality projections using survey-determined rates and methods approximating those used in the Kolbe et al. [7] and Schwartz et al. [8] studies are presented in Table 4. Using direct extrapolation, estimated total mortality for the affected population was 74,190 (range: 63,061-86,555). Extrapolation by damage level yielded a total estimate of 63,901 deaths (range: 49,033-81,862); a significant difference was observed between mortality rates of households living in homes that were mildly or moderately damaged compared to those that were significantly damaged or destroyed (p < 0.001)^a^.

**Table 4:** Earthquake mortality projections*

***Direct extrapolation to the exposed population***				
	2009 population^30^	Point estimate	Low estimate	High estimate
Survey rate (deaths per 1,000)	--	24.0	20.4	28.0
Metropolitan Port-au-Prince mortality	2,457,807	58,987	50,139	68,819
All affected areas mortality	3,091,236	**74,190**	**63,061**	**86,555**
***Extrapolation by damage level***				
***Survey mortality rates by residence damage Level (deaths per 1,000, 95% CI)***				
building destruction level category for pre-earthquake residence (respondent reported based on the MTMPC survey) [31]	Deaths/total exposed	Point estimate	Low estimate	High estimate
Green	11/531	20.7	10.4	36.8
Yellow	35/3216	10.9	7.6	15.1
Green and yellow (mild to moderate damage)	46/2847	16.2	11.9	21.5
Red (significant damage/destroyed)	107/2775	38.6	31.7	46.4
***Significantly damaged or destroyed residences***		N	Total	Percent
MTMPC Building Survey		77,674	382,256	20.3%
Remote-sensing damage assessment [29]				
Metropolitan Port-au-Prince		47,903	241,791	19.8%
All affected areas		59,073	299,257	19.7%
***Estimated number of deaths by residential destruction level***				
	Population estimate**	Point estimate	Low estimate	High estimate
**Metropolitan Port-au-Prince**				
Mild to moderate damage	1,966,246	31,853	23,398	42,274
Significant damage/destroyed	491,561	18,954	15,587	22,813
Total		50,807	38,986	65,088
**All affected areas**				
Mild to moderate damage	2,472,989	40,062	29,429	53,169
Significant damage/destroyed	618,247	23,839	19,605	28,693
Total		**63,901**	**49,033**	**81,862**

*The direct extrapolation approach is similar to the method used by Kolbe et al., while the extrapolation by damage level approach is similar to the method used by Schwartz and incorporates damage levels of residences. For all rates, the survey-determined point estimate and upper and lower bounds for confidence intervals are applied to the estimated population.

**Calculated by multiplying the 2009 population figures by 20.0% (proportion of residences significantly damaged or destroyed) and 80% (residences not significantly damaged or destroyed).

## Discussion

There is substantial variation in earthquake mortality outcomes [9]. Low levels of economic development have been associated with higher earthquake mortality, suggesting that poorer countries face increased risk due to a variety of characteristics of the built environment [9-11]. Building collapse accounts for almost all earthquake-related death, with a majority of deaths occurring indoors [9,11-13]. Construction materials that have been associated with increased mortality or injury risk include unreinforced masonry[14], mud and stone walls [15], concrete [16], panel construction [17], and wood [18-20].Worldwide, the greatest risk of collapse and resulting deaths is from unreinforced masonry structures (including mud brick) [21]. Other features of the built environment and individual location (at the time of the earthquake) associated with increased risk for earthquake mortality include being on the ground floor [22], or upper floors [11,23] of multistory buildings, earthquake intensity, and distance to the epicenter [24-27]. Individual-level characteristics associated with increased risk for earthquake mortality and injury include both age and sex. Higher rates of death among older populations are common [17,23,26-33] and several studies also show that children face an increased risk of death [15,25,27,31]. Female sex was significantly associated with increased risk of death in at least three earthquakes [10,29,32]; however, a similar number of studies found no significant difference in mortality by sex [24,25,28,32].

This analysis is one of several attempts to characterize mortality following the 2010 Haitian earthquake. The results from the data collected in this study found an earthquake mortality rate of 24 (CI: 20–28) deaths per 1,000 in the sample population. This observation suggests that mortality was nearly four-fold lower than officially reported figures. At one year after the earthquake, UN revised mortality figures estimated that 222,750 deaths occurred [34]. With a population of 3.1 million in the affected areas [6], this equates to a mortality rate of approximately 72 deaths per 1,000. The methods by which the official mortality estimates were obtained are undocumented, and their accuracy has been questioned, with some critics suggesting they are inflated, in particular given the substantially lower casualty figures reported in the immediate aftermath [8]. However, our study could underestimate the total deaths because the sampling did not account for households with high or complete mortality that could not be sampled. Other scientific studies have estimated Haitian earthquake mortality at 111,794 (CI: 93,273–130,316) deaths during or immediately after the earthquake, with a mortality rate of 41 (CI: 35–48) deaths per 1,000 [7] and between 46,190 and 84,961 deaths, with a mortality rate of 22 deaths per 1,000 [8].

Extrapolation of survey-based rates to large urban populations in instances such as Haiti where damage and mortality are highly variable and geographically concentrated presents numerous methodological challenges, particularly in the context of widespread displacement and mortality. While data from three epidemiologic studies conducted to date clearly indicate lower levels of mortality than the official estimates, there are noteworthy differences in study methods that likely contribute to the observed variation in mortality estimates. The Kolbe et al. study [7] used a pre-earthquake random sample of Port-au-Prince households and then sought to follow up the population postearthquake, whereas this survey and the Schwartz study [8] intended to capture representative samples of the affected population. The extrapolation methods also differed with Kolbe et al., using direct extrapolation of the survey rate to the affected population, whereas the Schwartz et al. and the present study determined mortality rates by building damage level to account for variation in impacts.

While both extrapolation methods were applied in the current paper, the authors believe the methodology that takes damage level into account is more accurate. When damage levels are accounted for, the results of the present study yielded an estimate of 63,901 deaths (range: 49,033-81,862), which is consistent with the Schwartz estimate of 46,190 to 84,961 deaths. Extrapolating from structure damage levels could underestimate fatalities because there is no distinction within the “red” category (uninhabitable) between damaged but standing and collapsed buildings, despite structural collapse being the greatest predictor of mortality [20,24]. While these values provide another independent projection of the number of earthquake fatalities, it is important to note that other more advanced methods of mortality modeling, including those that incorporate Geographic Information Systems (GIS) and which are based on a larger sample size, would likely yield more accurate estimates of total mortality.

Age was important in predicting the odds of death in this study, with increased mortality risk observed among older adults, a finding common to many earthquake settings [17,22,25-32]. However, this observation is contrary to the Kolbe et al. study [7], which found that children were 5.8 (CI: 4.0-8.3) times more likely to have been killed in the earthquake than adults, and that children accounted for the majority of earthquake deaths. Unfortunately, no other findings on age or sex as a risk factor for death in the Haitian earthquake have been reported, which limits further comparison. In this study, the population displaced to camps had a higher mortality rate compared to those remaining in neighborhoods; however, the statistical significance of this finding was marginal. Number of levels in the housing structure and crowding, a proxy for both socioeconomic status and population density, were both associated with mortality risk. In our analysis, residential construction characteristics such as roof or wall construction material, housing type (apartment, detached, or attached home), and number of levels, had varying levels of association with mortality in simple regression, and only the number of levels was included in the final multiple regression model. Associations between single versus multilevel homes, crowding, and increased mortality risk support the hypothesis that earthquake vulnerability is related to the built environment and that households of lower socioeconomic status with high levels of population density face increased risk of both displacement and death.

The high levels of mortality observed in Haiti were largely attributable to substandard and unsupervised construction practices [35]. Given that most mortality and injury following earthquakes is mediated by building damage, rebuilding structures with improved earthquake-proof construction is critical for future disaster preparedness efforts. Substantial investment in infrastructure, urban planning, and building code enforcement are required if reconstruction efforts are to yield sound earthquake-resilient structures. However, mitigating the risk posed by poor construction is especially challenging and perhaps unfeasible in resource-poor settings where retrofitting buildings and code enforcement are both cost-prohibitive and often not a priority for local authorities and at risk populations.

### Limitations

Sample size calculations for the survey were based on initial mortality reports from the Haitian government; however, observed mortality was three-fold lower than rates used for sample planning. A larger sample size would have improved exploration of risk factors, detection of significant differences, and led to greater precision. One limitation of cluster survey designs in earthquake settings is that areas of concentrated deaths, such as collapsed apartment buildings, are likely to be missed, which could result in the underestimation of mortality. Another limitation that likely contributed to the underestimation of mortality is survivor bias, in which households where no members survived or where few members survived were not included in the retrospective mortality estimates. Furthermore, it is possible that the sample was not representative of the affected population because replacement sampling was used, when households that were not at home were systematically excluded. With respect to assessing risk factors for death, improved descriptions of buildings in which deaths occurred and location of individuals at the time of the earthquake would have been useful in analyzing the built environment’s mediating effect on mortality outcomes. However, the long recall period, potential inaccuracies in reporting relevant environmental details for the pre-earthquake residence location of all household members, and the inability to systematically confirm reported building material data for the majority of individuals prohibited the collection of more detailed information on the built environment and subsequent risk analysis. Finally, more research is needed to investigate how built environment characteristics can or cannot serve as proxies for socioeconomic status; a clear understanding of these relationships would have enabled stronger conclusions.

## Conclusion

Estimation of mortality and morbidity in natural disasters and conflicts is controversial, and inconsistencies in the reported impacts of these events are not uncommon. The importance of death and injury counts for humanitarian response planning, documentation of human rights violations, and longer-term public policy responses to emergencies is increasingly accepted. However, the methods used to arrive at these estimates are often poorly documented, widely variable, and often the subject of intense debate criticism. Mortality in the 2010 Haitian earthquake is no exception. Results of epidemiologic surveys suggest a substantially lower mortality burden than figures reported by the Haitian government and United Nations. Findings from this study indicate an earthquake mortality rate of 24 deaths per 1,000 (CI: 20–28) in the metropolitan Port-au-Prince survey population. The most important predictors of mortality risk were older age, residence in a multilevel structure, high levels of crowding, and location at the time of the earthquake. A better understanding of risk factors associated with earthquake mortality and the risk attributed to the built environment is essential for humanitarian response planning and to implement risk reduction strategies that are feasible in resource-poor settings.

## Consent

Respondents were read a statement describing the survey and requesting their participation, and oral consent was obtained prior to initiating the interview.

## Endnotes

^a^Ministry of Transportation and Public Works Building Damage Assessment result for the residence as reported by respondents in the household survey.

## Competing interests

The authors declared that they have no competing interest.

## Authors’ contributions

SD: Principal Investigator; Study Design; Oversight of Data Collection; Writing. TK: Critical Analysis and Review; Writing. MN: Data Analysis; Writing. All authors read and approved the final manuscript.
